# Taiman acts as a coactivator of Yorkie in the Hippo pathway to promote tissue growth and intestinal regeneration

**DOI:** 10.1038/celldisc.2016.6

**Published:** 2016-03-22

**Authors:** Chao Wang, Meng-Xin Yin, Wei Wu, Liang Dong, Shimin Wang, Yi Lu, Jinjin Xu, Wenqing Wu, Sheng Li, Yun Zhao, Lei Zhang

**Affiliations:** 1 State Key Laboratory of Cell Biology, CAS Center for Excellence in Molecular Cell Science, Innovation Center for Cell Signaling Network, Institute of Biochemistry and Cell Biology, Shanghai Institutes for Biological Sciences, Chinese Academy of Sciences, Shanghai, China; 2 Key Laboratory of Insect Developmental and Evolutionary Biology, Institute of Plant Physiology and Ecology, Shanghai Institutes for Biological Sciences, Chinese Academy of Sciences, Shanghai, China; 3 School of Life Science and Technology, ShanghaiTech University, Shanghai, China

**Keywords:** Hippo pathway, proliferation, regeneration, Taiman, Yorkie

## Abstract

The Hippo signaling pathway regulates tissue growth and organ size through controlling cell growth, proliferation and apoptosis. During these processes, the coactivator Yorkie partners with the transcription factor Scalloped to mediate Hippo pathway-regulated cellular functions. Here, we demonstrate that Taiman facilitates the activity of Yorkie. First, Taiman overexpression upregulates Hippo pathway-responsive genes and induces tissue overgrowth. Second, the loss of *tai* downregulates the expression of Hippo pathway target genes and reduces organ size as well as tissue overgrowth caused by Yorkie overexpression. Furthermore, we provide evidence that Taiman binds to Yorkie and facilitates the activity of Yorkie-Scalloped to activate the transcription of several Hippo pathway target genes. Moreover, we found that the C-terminus of Taiman is indispensable for the function of Taiman in Hippo signaling. Finally, we demonstrate that Taiman is also required in intestinal stem cell proliferation. Our findings suggest Taiman is an essential coactivator of Yorkie.

## Introduction

Organ size control is a highly refined biological process, which is fundamental for multicellular life [1]. In this developmental process, cell growth, cell proliferation and cell apoptosis are delicately coordinated; yet the underlying mechanisms are not completely clarified. Previous studies have identified the Hippo pathway as a crucial mechanism controlling this process [2, 3]. Initially discovered in *Drosophila*, the Hippo signaling pathway has emerged as a central and highly conserved pathway that regulates tissue growth and organ size [1, 3]. Moreover, accumulating evidence has suggested that the Hippo pathway also has an important role in tissue homeostasis, tissue repair and even stem cell maintenance [4–7]. Especially, the Hippo pathway has an essential role in regulating *Drosophila* adult intestinal stem cell (ISC) proliferation [7, 8]. Malfunctions of Hippo signaling are closely related to a wide variety of human cancers and diseases [1].

A crucial aspect of research on Hippo signaling is the regulation of Yorkie (Yki) activity as the upstream signals converge on Yki eventually [6]. Yki and the transcription factor Scalloped (Sd) act together to regulate Hippo pathway target genes [9, 10]. Several recent studies have reported novel regulators of Yki, including Mask, Brahma and Ncoa6, which shed some light on Yki-mediated transcriptional regulation [11–14]. These cofactors interact with Yki and are necessary for Yki’s activity. However, our understanding of the regulation of Yki’s activity remains incomplete.

Taiman (Tai) was first reported as a coactivator of Ecdysone Receptor (EcR). Much of what we know about Tai comes from studies using *Drosophila* ovary. Tai cooperates with EcR to maintain the normal migration of the border cells [15]. AIB1, the mammalian homolog of Tai, is reported to be closely related to a wide range of human cancers, suggesting that it might have an important role in cell growth control [16–18]. Recently, Zhang *et al.* reported that Tai and Yki collaboratively induce germline genes in developing somatic epithelia to regulate tissue growth [19].

In the current study, we independently identify Tai as a coactivator of Yki through a genetic screening for new modulators of the Hippo pathway. We provide evidence that Tai overexpression upregulates Hippo pathway-responsive genes and induces tissue overgrowth. We show that the loss of Tai not only downregulates Hippo pathway target genes but also reduces organ size as well as tissue overgrowth caused by Yki overexpression. Furthermore, we provide evidence that Tai binds to Yki and facilitates the activity of Yki-Sd to activate the transcription of several Hippo pathway target genes. Moreover, C-terminus of Tai is found to be indispensable for the function of Tai in Hippo signaling. In addition, we demonstrate that Tai is also required in both dextran sulfate sodium (DSS)-induced and Yki-induced ISC proliferation.

## Results

### Identification of *tai* as a gene positively regulating tissue growth

**Figure 1:**
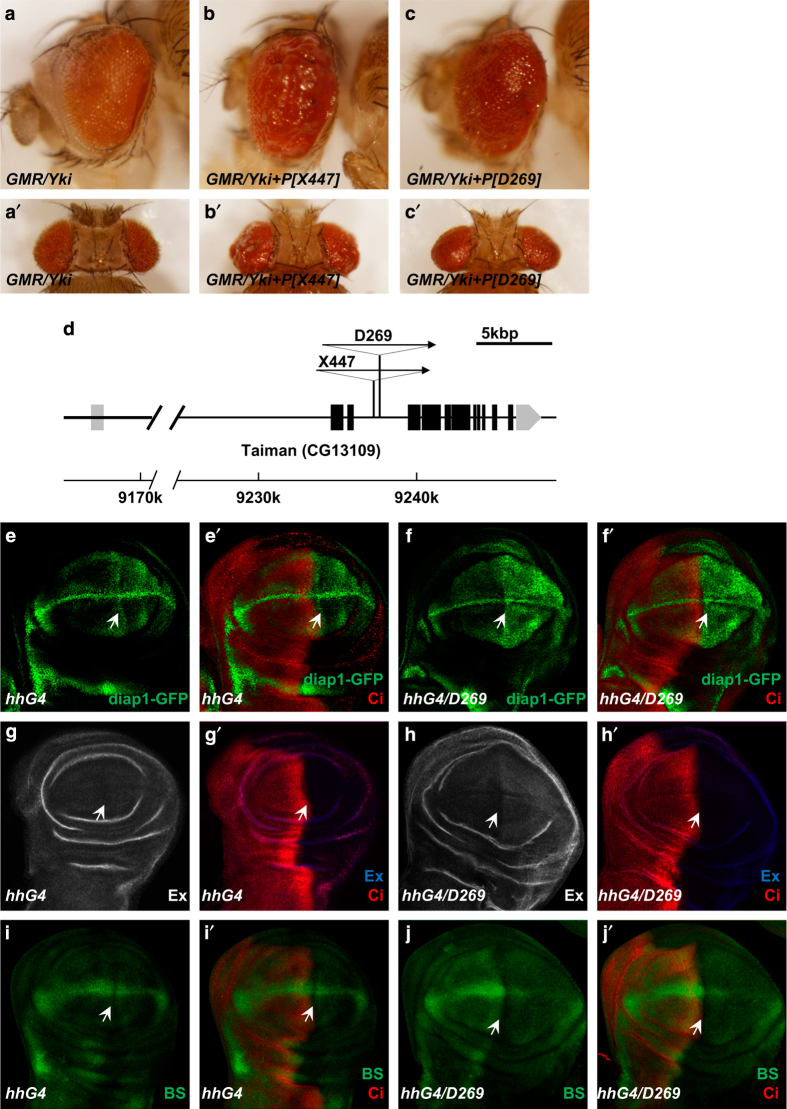
Tai P-element insertion lines trigger tissue growth and enhance the Hippo pathway target gene expression. (**a**–**c**′) Side (**a**–**c**) and dorsal (**a**′–**c**′) views of adult fly eyes expressing *UAS-Yki* (**a**, **a**′), *UAS-Yki+*[X447] (**b**, **b**′) or *UAS-Yki+*[D269] (**c**, **c**′) with *GMR-Gal4*. Scale bars, 100 μm. (**d**) Schematic representation of the *tai* gene locus and P-element insertion sites. The splicing pattern of the major *tai* product is indicated, as are the sites of insertion of the P-element alleles. The predicted coding sequence is shaded. The insertion sites of [D269] and [X447] EP lines are in the third intron of *tai* gene. (**e**–**j**′) Control wing discs (**e**, **e**′) or wing discs expressing [D269] with *hh-Gal4* (**f**, **f**′) were immunostained to show the expression of *diap1-GFP* (**e**–**f**′), Ex (**g**–**h**′) and *bantam-GFP* (BS) (**i**–**j**′). Arrows indicate the P-compartment. Scale bars, 50 μm.

To identify novel components of the Hippo pathway, we performed a genetic screen in which flies carrying *GMR-Gal4* and *UAS-Yki* (referred to as *GMR-Yki*) were crossed with a collection of EP lines. The overgrown eye phenotype induced by overexpression of Yki provides a sensitive background for us to identify modulators of the Hippo pathway. Through screening the progeny of *GMR-Yki* and more than 10 000 EP lines, we found numerous enhancers and suppressors that can further enhance the eye overgrowth induced by Yki overexpression. We then analyzed the UAS element insertion sites of these lines for new candidates. In this screen, we found two EP lines ([X447], [D269]) that have the *UAS* element insertion in an intron of *tai* gene that could dramatically promote the overgrown phenotype of *GMR-Yki* (Figure 1a-d).

To further determine whether the expression of *tai* EP line could affect the activity of Hippo pathway, [D269] was expressed under the control of *hh-Gal4* driver that drives gene ectopically expressed in the posterior compartment (P-compartment) of wing imaginal discs. Then, the changes in expression levels of well-characterized Hippo pathway target genes (*diap1, ex, bantam*) were examined (Figure 1e-j′). As shown in Figure 1f-f′ and h-h′, the expression of [D269] led to the upregulation of diap1-GFP and ex. Meanwhile, the expression of a *bantam* sensor (BS), *bantam-GFP*, which inversely indicates the bantam microRNA level, was reduced by [D269] overexpression (Figure 1j-j′). Briefly, the overexpression of [D269] upregulated expression levels of the Hippo pathway target genes. The results presented above suggest that the expression of [D269] promotes tissue growth and influences Hippo signaling.

### Tai overexpression induces tissue growth through Hippo signaling

**Figure 2:**
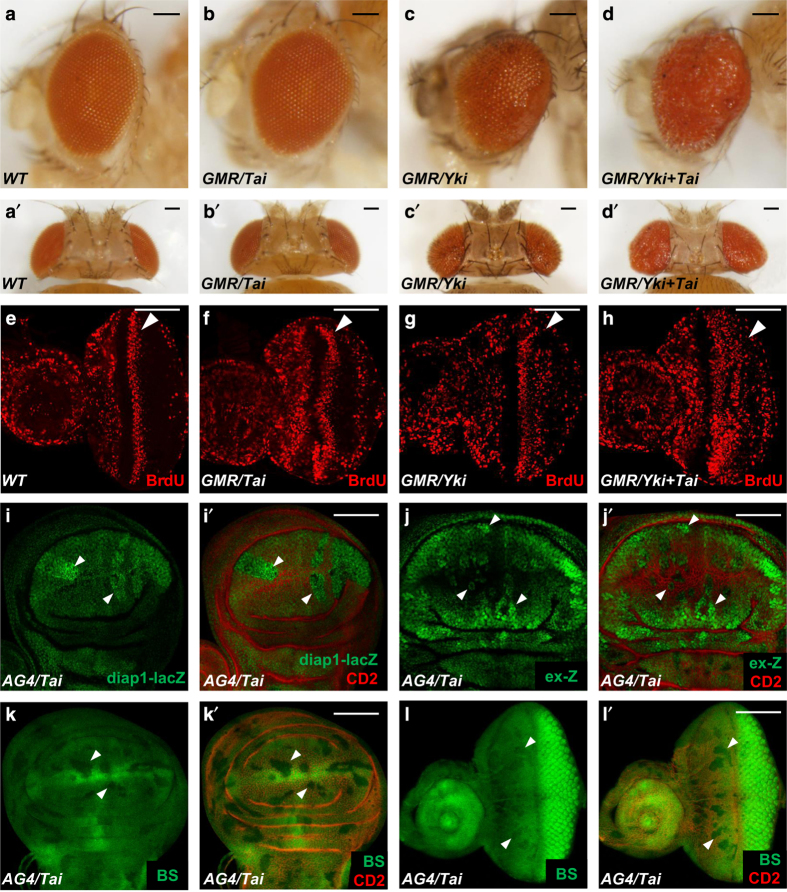
Overexpression of Tai promotes tissue overgrowth induced by Yki and activates the expression of Hippo pathway target genes. (**a**–**d**′) Side (**a**–**d**) and dorsal (**a**′–**d**′) views of wild-type adult eye (**a**, **a**′), eyes expressing *UAS-Tai* (**b**, **b**′), *UAS-Yki* (**c**, **c**′) and *UAS-Yki+UAS-Tai* (**d**, **d**′) with *GMR-Gal4*. Scale bars, 100 μm. (**e**–**h**) BrdU incorporation in wild-type eye discs (**e**), eye discs expressing *UAS-Tai* (**f**), *UAS-Yki* (**g**), or *UAS-Yki+UAS-Tai* (**h**) with *GMR-Gal4*. Arrowheads indicate the position of second mitotic wave. Scale bars, 50 μm. (**i**–**l**′) *Drosophila* discs containing flip-out clones expressing *UAS-Tai* by *act>CD2>Gal4* (*AG4*) were immunostained to show the expression of *diap1-lacZ* (**i**, **i**′), *ex-lacZ* (*ex-Z*) (**j**, **j**′) or *bantam-GFP* (*BS*) (**k**–**l**′). Clones expressing *UAS-Tai* were marked by the lack of CD2 staining and indicated by arrowheads. Scale bars, 50 μm.

To verify the functional relationship between *tai* and the Hippo pathway, we generated *UAS-Tai* transgenic flies. Consistent with the results in Figure 1, coexpression of Tai and Yki enhanced the eye overgrowth induced by Yki overexpression, though overexpression of Tai alone did not induce obvious overgrowth phenotype (Figure 2a-d′). In addition, the overexpression of Tai dramatically enhanced the increase of ectopic BrdU incorporation caused by *GMR-Yki* posterior to the second mitotic wave (Figure 2e-h), indicating a proliferation of boosted cells.

We then expressed Tai in imaginal discs using *Actin>CD2>Gal4* (referred as *AG4*) and examined the changes of expression levels of Hippo pathway target genes. As shown in Figure 2i-j′, overexpression of Tai induced a dramatic increase of *diap1-lacZ* (Figure 2i-i′) and *ex-lacZ* (ex-Z) (Figure 2j-j′) and obvious decrease of *bantam-GFP* (Figure 2k-l′), suggesting that the overexpression of Tai promotes the expression of Hippo pathway target genes. These findings confirm that overexpression of Tai induces cell proliferation and tissue overgrowth by activating the transcription of Hippo pathway target genes.

### Loss of *tai* inhibits tissue growth by downregulation of Hippo pathway targets

**Figure 3:**
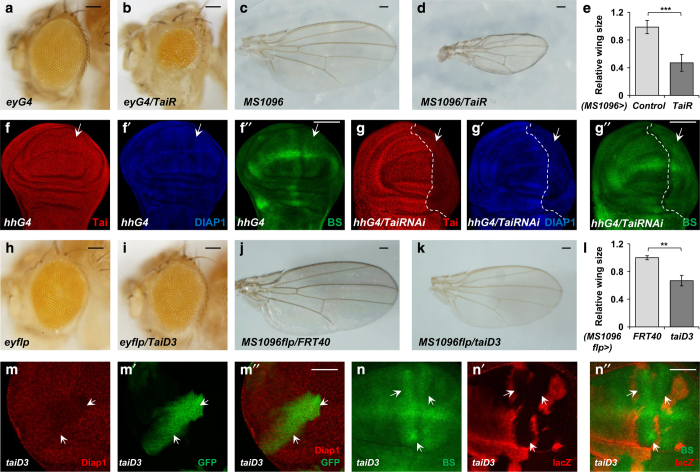
Loss of Tai inhibits tissue growth and suppresses the expression of Hippo pathway target genes. (**a**, **b**) Side views of eyes from control adult fly (**a**) or fly expressing *Tai-RNAi* (*TaiR*) (**b**) under the control of *eyeless-Gal4*. Scale bars, 100 μm. (**c**, **d**) Dorsal view of wings from control adult fly (**c**) or fly expressing *Tai-RNAi* (**d**) with *MS1096-Gal4*. Scale bars, 250 μm. (**e**) The relative wing size comparing control wings (**c**) and wings expressing *Tai-RNAi* (**d**). The data were quantified using an unpaired *t*-test. The results represented the mean± s.e.m. ***P<0.001 (*n*>6) for each genotype. (**f**–**g**′′) Control wing discs (**f**–**f**′′) or wing discs expressing *Tai-RNAi* with *hh-Gal4* (**g**–**g**′′) were immunostained to show the expression of Tai (**f**, **g**), DIAP1 (**f**′, **g**′) or *bantam-GFP* (**f**′′, **g**′′). The white dashed lines mark the boundary between anterior and posterior parts in wing discs. Arrows indicate the P-compartment. Scale bars, 50 μm. (**h**, **i**) Side views of adult eyes containing control clones (**h**) or *taiD3* clones (**i**) induced by *ey-Flp*. Scale bars, 100 μm. (**j**, **k**) Dorsal views of adult wings containing control clones (**j**) or *taiD3* clones (**k**) induced by *MS1096-Flp*. Scale bars, 250 μm. (**l**) The relative wing size comparing control wings (**j**) and wings containing *taiD3* clones (**k**). The data were quantified using an unpaired *t*-test. The results represented the mean± s.e.m. **P<0.01 (*n*>6) for each genotype. (**m**–**m**′′) A large magnification view of eye discs carrying *taiD3* mutant clones that immunostained to show the expression of DIAP1 (**m**, **m**′′). Clones are marked by GFP (**m**′) and indicated by the arrows. Scale bar, 25 μm. (**n**–**n**′′) Wing discs containing *taiD3* mutant clones were immunostained to show the expression of *bantam-GFP* (**n**, **n**′′). Mutant cells are marked by the lack of lacZ expression (**n**′) and indicated by arrows. Scale bar, 25 μm.

To further assess the role of Tai in tissue growth and the regulation of Hippo target gene expression, we generated two RNAi transgenes targeting two different regions of *tai* (Supplementary Figure S1A). Both RNAi transgenes effectively knocked down endogenous Tai (Supplementary Figure S1B-C). Expression of *UAS-Tai-RNAi* by the *eyeless-Gal4* (*eyG4*) driver, which drives gene to express in eye discs, resulted in a significant decrease of *Drosophila* eye size (Figure 3b compared with Figure 3a). In addition, expression of *UAS-Tai-RNAi* using the wing-disc-specific driver *MS1096-Gal4* (*MS1096*) induced an obvious decrease in wing size (Figure 3c-e). These observations suggest that Tai is necessary for normal eye and wing development.

We further tested whether the knockdown of Tai could result in downregulation of the expression of Hippo pathway target genes. As shown in Figure 3f′ and g′ and Supplementary Figure S1E′, Tai knockdown led to the decrease of *diap1* ectopic expression and *ex-lacZ*. The expression of *bantam* microRNA was also suppressed by Tai knockdown that was indicated by an increase of *bantam-GFP* (compare Figure 3g′′ with f′′). These results suggest that Tai is crucial for normal tissue growth and the regulation of Hippo pathway target genes.

To further strengthen the conclusion from RNAi experiments, we generated *tai* mutant with CRISPR/Cas9 strategy (Supplementary Figure S1F-H). The deletion in *taiD3* mutant leads to a stop codon in the N-terminus (Supplementary Figure S1F and G). In *taiD3* homozygous clones generated using Mosaic Analysis with a Repressible Cell Marker system, endogenous Tai can hardly be detected (Supplementary Figure S1H). Meanwhile, the heterozygous progeny of *taiD3* and another validated tai mutant *tai* ^*61G1*^, *taiD3/tai* ^*61G1*^, were lethal. These results suggest that *taiD3* is a reliable mutant for further experiments.

We then determined whether tai mutant could suppress normal tissue growth. Using the *eyeless-flipase* (*eyflp*) and *MS1096-flipase* (*MS1096flp*), we generated *taiD3* homozygous clones in *Drosophila* eyes and wings, respectively. As shown in Figure 3h-l, the loss of *tai* suppressed *Drosophila* tissue growth. Correspondingly, we checked whether the loss of *tai* leads to a downregulation of Hippo pathway target gene expression. Of note, in *taiD3* mutant clones, Diap1 protein level was decreased (Figure 3m-m′′). In addition, *bantam* microRNA level, indicated by the upregulation of *bantam-GFP*, was reduced in *taiD3* mutant clones (Figure 3n-n′′). All these findings further implicate that Tai regulates the expression of Hippo pathway-responsive genes and functions in growth control.

### Tai acts downstream of the Hippo pathway

**Figure 4:**
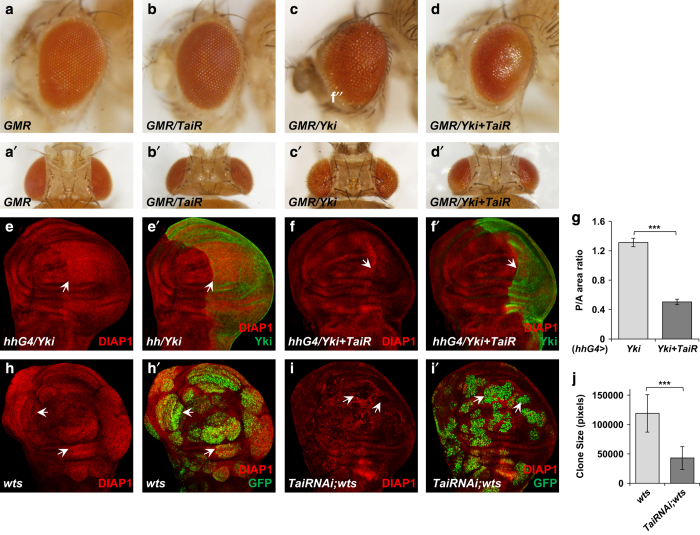
Genetic interaction between *tai* and the Hippo pathway components. (**a**–**d**′) Side (**a**–**d**) and dorsal (**a**′–**d**′) views of control adult fly eyes or eyes expressing *Tai-RNAi* or *UAS-Yki* or *UAS-Yki+Tai-RNAi* under the control of *GMR-Gal4*. Scale bars, 100 μm. (**e**–**f**′) Wing discs expressing *UAS-Yki* (**e**, **e**′) or *UAS-Yki+Tai-RNAi* (**f**, **f**′) with *hh-Gal4* were immunostained to show the expression of DIAP1. The P-compartment is indicated by Yki staining (**e**′, **f**′) and arrows. Scale bars, 50 μm. (**g**) The comparison of P/A area ratio between wing discs shown in **e**, **f**. The data were quantified using an unpaired *t*-test. The results represented the mean± s.e.m. ***P<0.001 (*n*>6) for each genotype. (**h**–**i**′) Wing discs containing Mosaic Analysis with a Repressible Cell Marker (MARCM) clones of wts mutant (**h**, **h**′) and wts mutant with *Tai-RNAi* (V15709) (**i**, **i**′) were stained for DIAP1. Clones are marked by GFP and indicated by arrows. Scale bars, 50 μm. (**j**) The comparison of the size of MARCM clones shown in **h**, **i**. The data were quantified using an unpaired *t*-test. The results represented the mean± s.e.m. ***P<0.001 (*n*>6) for each genotype.

According to the observations demonstrated in previous sections, we next determined the functional relationship between Tai and the components of Hippo signaling pathway through genetic analysis. During our experiments, it is noted that coexpression of *UAS-Tai-RNAi* with *UAS-Yki* reduced the eye overgrowth induced by Yki overexpression (Figure 4a-d′), suggesting that *tai* may act downstream of the Hippo pathway. To verify our presumption, we investigated the changes of the expression of Hippo pathway target genes in imaginal discs. Expression of Yki by the *hh-Gal4* driver resulted in a dramatic upregulation of DIAP1 protein and induced an overgrowth of P-compartment in wing discs (Figure 4e-e′). Depletion of Tai using RNAi suppressed the elevated DIAP1 expression and the overgrowth induced by Yki (Figure 4f and g). In addition, we found that *tai*^*61G1*^ heterozygote reduced the eye overgrowth induced by Yki overexpression and enhanced the scalloped wing phenotype caused by *Sd*^*1*^ (Supplementary Figure S2A-E). Furthermore, we utilized the Mosaic Analysis with a Repressible Cell Marker system to examine the genetic interaction between *tai* and the Hippo pathway. As shown in Figure 4h-h′, *warts* (*wts*) mutant clones led to significant tissue overgrowth and elevated DIAP1 expression. Tai knockdown suppressed the overgrowth and reduced DIAP1 expression in *wts* mutant clones (Figure 4i and j). These results further support the notion that *tai* functions downstream of *wts* in the Hippo pathway.

### Tai is essential for Yki’s activity and functions in parallel to Yki in Hippo signaling

**Figure 5:**
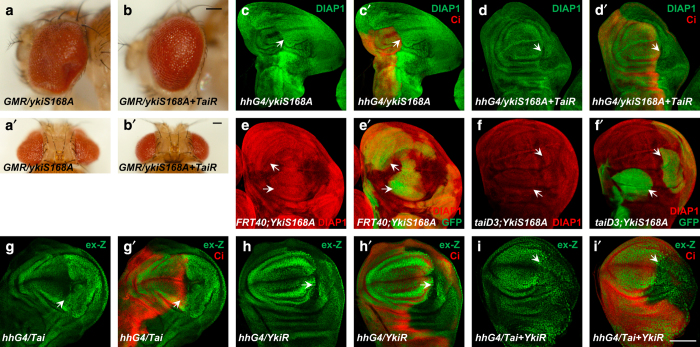
Tai is required for the activity of Yki and functions in parallel to Yki in the Hippo pathway. (**a**–**b**′) Side (**a**, **b**) and dorsal (**a**′, **b**′) views of eyes expressing *YkiS168A* (**a**, **a**′) or *YkiS168A+Tai-RNAi* with *GMR-Gal4*. Scale bars, 100 μm. (**c**–**d**′) Wing discs expressing *YkiS168A* (**c, c**′) or *YkiS168A+Tai-RNAi* (**d**, **d**′) under the control of *hh-Gal4* were immunostained to show the expression of DIAP1. Ci marks the A-compartment and arrows indicate the P-compartment. (**e**–**f**′) Wing discs containing Mosaic Analysis with a Repressible Cell Marker clones of *taiD3* mutant (**e**, **e**′) and *taiD3* mutant with YkiS168A (**f, f**′) were stained for DIAP1. Clones are marked by GFP and indicated by the arrows. (**g**–**i**′) Wing discs expressing *Tai* (**g**, **g**′) or *YkiRNAi* (*YkiR*) (**h**, **h**′) or *Tai+YkiRNAi* (**i, i**′) under the control of *hh-Gal4* were immunostained to show the expression of *ex-lacZ*. Ci marks the A-compartment and arrows indicate the P-compartment. Scale bars, 100 μm.

Given that *tai* functions downstream of *wts*, we next investigated how loss of tai compromised the activity of Yki using genetic analysis. To eliminate the interference of the activity change of the Hippo pathway, we took advantage of Yki active variant YkiS168A, which is not refrained by upstream Hippo pathway signals. As shown in Figure 5a-b′, depletion of Tai by RNAi repressed the *Drosophila* eye overgrowth induced by YkiS168A overexpression. Tai knockdown also suppressed the overgrowth as well as the increased DIAP1 expression caused by YkiS168A in wing discs (Figure 5c-d′). Moreover, Tai RNAi rescued the decrease of *bantam-GFP* induced by Yki overexpression (Supplementary Figure S2F-H′). In addition, *taiD3* limited the overgrowth in clones as well as the increased DIAP1 expression induced by excessive YkiS168A (Figure 5e-f′). The observations above suggest that Tai is necessary for Yki’s activity. It is also worth noting that, although depletion of Tai could inhibit tissue overgrowth induced by Yki overexpression, it could not block some elevated Hippo target genes, such as *ex-lacZ* (Supplementary Figure S2I-J′). It implies that Tai may not be the only coactivator required for the function of Yki.

Furthermore, we examined whether Yki is dispensable for the function of Tai in Hippo signaling. As shown in Figure 5g-i′, expression of Tai induced a significant increase of *ex-lacZ* while coexpression of *UAS-Yki-RNAi* reduced *ex-lacZ* as well as wing disc size. These results indicate that Yki is indispensable for the function of Tai in the Hippo pathway. Taken together, Tai is essential for Yki’s activity and *tai* functions in parallel to *yki* in Hippo signaling.

Considering that Yki primarily binds to Sd to form transcription complex in Hippo signaling, we tested the functional relationship between *sd* and *tai*. Sd knockdown suppressed the elevated *diap-GFP* level caused by overexpression of Tai, suggesting that Sd is necessary for the function of Tai (Supplementary Figure S2K-M′). Interestingly, we found that the eye overgrowth induced by the expression of constitutively active Sd (SdGA) was not affected by Tai knockdown (Supplementary Figure S2N-O′). Consistently, Tai knockdown did not reduce the increase of *diap1-lacZ* expression induced by SdGA (Supplementary Figure S2P-Q′). These findings imply that *tai* functions upstream of *sd* and further strengthen that *tai* and *yki* work in parallel.

### Tai interacts with Yki and facilitates the activity of Yki-Sd complex

**Figure 6:**
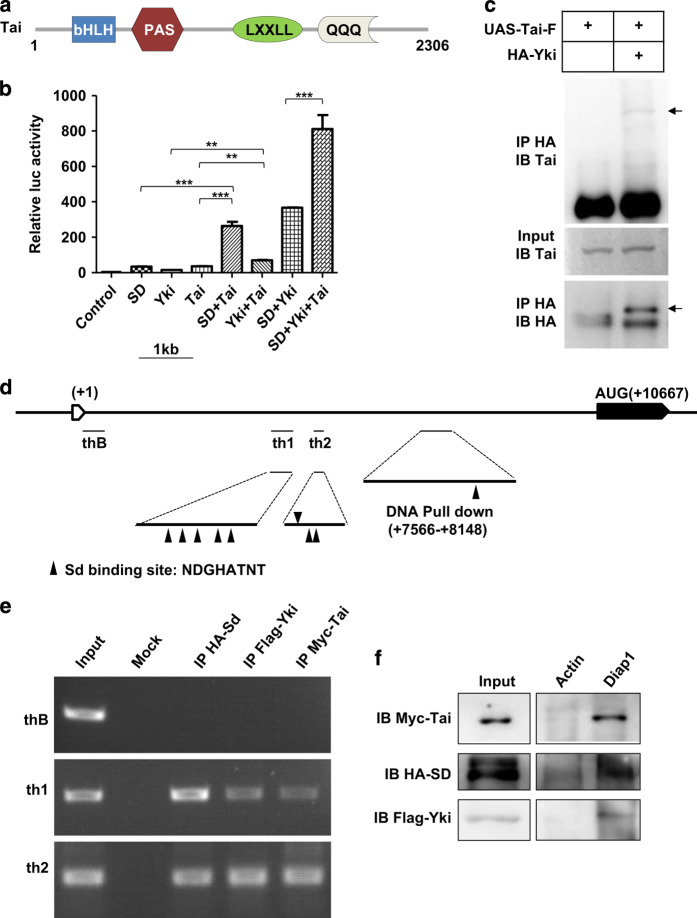
Tai binds to Yki and forms a complex with Sd-Yki on target gene promoter. (**a**) Schematic diagram of the Tai protein domain structure. (**b**) Tai acts synergistically with Sd-Yki to activate *3xSd2-luc* reporter gene. S2 cells were transfected with indicated constructs and luciferase reporter gene, followed by dual luciferase assay. The data were quantified using an unpaired *t*-test. The results represented the mean± s.e.m. ***P<0.001 (*n*=3). (**c**) CoIP of Tai with HA-tagged Yki. S2 cells were transfected with the indicated Tai and Yki constructs, followed by immunoprecipitation and western blot analysis with the indicated antibodies. The specific bands were indicated by arrows. (**d)** Diagram of the *diap1* promoter. Fragments for ChIP analysis and DNA pull-down are marked. Sd-binding motifs were indicated by arrowheads. th1 and th2 stand for the two regions containing several Sd-binding motifs. thB is the negative control. The biotin-tagged DNA fragment (7566-8148) also contains Sd-binding motif. (**e**) Tai binds to the promoter of *diap1*. Cells were transfected with *HA-Sd*, *Flag-Yki* and *Myc-Tai*, followed by ChIP assay with indicated antibodies. HA-Sd was added in as a positive control. (**f**) Tai, Sd and Yki bind to *diap1* DNA sequences but not to actin. DNA pull-down with either actin or *diap1* DNA sequences from extracts of S2 cells transfected with *Myc-Tai*, *Flag-Yki* and *HA-Sd*.

Tai protein was first reported as a coactivator of EcR. It contains four major domains: a basic helix-loop-helix (bHLH), a PAS domain, LxxLL motifs and domain containing several polyglutamine stretches (PolyQ) (Figure 6a). To probe the molecular mechanism by which Tai functions in Hippo signaling, we performed several assays in S2 cells. In a dual luciferase assay, we used 3x*Sd2-Luc* reporter to reflect the transcriptional activity of Sd-Yki. We found that expression of Tai significantly enhanced the luciferase reporter activity triggered by coexpression of Sd and Yki (Figure 6b). This finding suggests that Tai activates the Sd-Yki transcriptional complex *in vitro*. Furthermore, as we noted, a published work implicated that Tai is likely to be a binding partner of Yki [20]. A recent work showed that Tai binds to Yki through PPxY motifs and WW domains [19]. We verified the interaction between Tai and Yki by coimmunoprecipitation experiment. As shown in Figure 6c, Tai was coimmunoprecipitated with Yki. We also performed chromatin immunoprecipitation (ChIP)-PCR experiment and DNA pull-down experiment using S2 cells. Yki, Sd and Tai were cotransfected in S2 cells and then cells were collected for the following assays. Moreover, ChIP-PCR analysis showed that Tai binds to the promoter region of diap1 as the same as Sd does (Figure 6d and e). In addition, the biotin-tagged DNA fragment from the *diap1* promoter pull-down Tai as well as Yki and Sd (Figure 6d and f). The results presented above supported the assumption that Tai interacts with Yki and facilitates the activity of Sd-Yki transcriptional complex.

### C-terminus of Tai is essential for the function of Tai in regulating Hippo signaling

**Figure 7:**
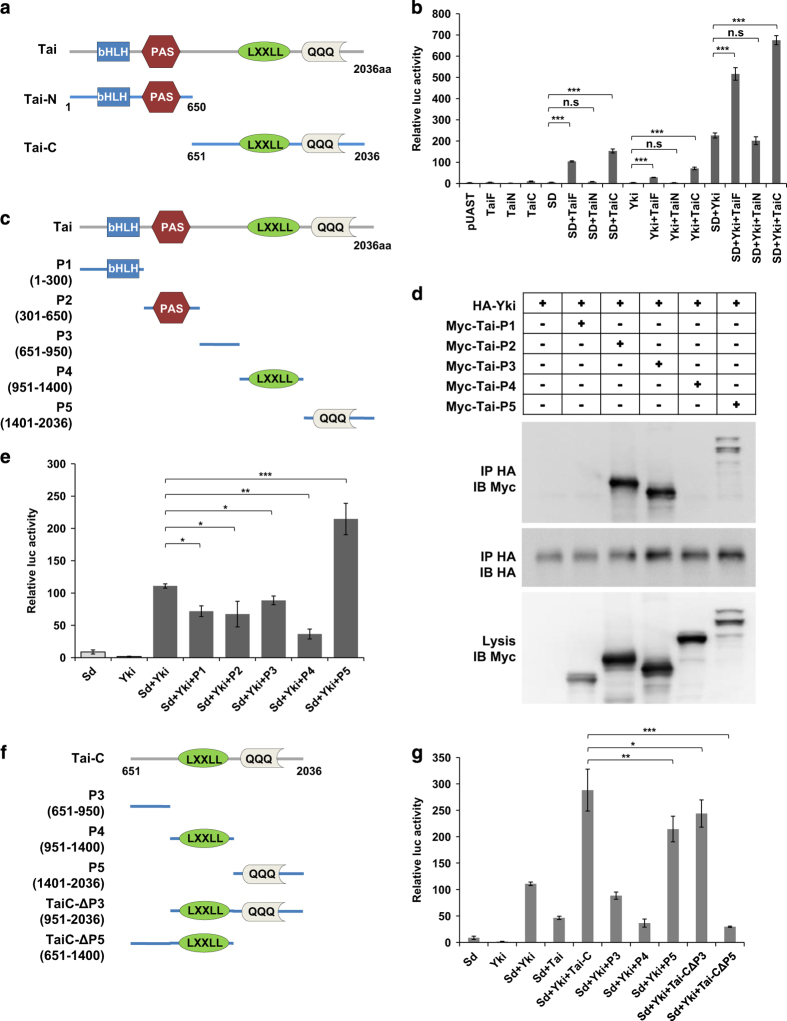
Characterization of different domains of Tai. (**a**) Schematic diagram of the N- and C- truncations of Tai protein. (**b**) TaiC sufficiently enhances the activity of Sd-Yki to trigger *3xSd2-luc* reporter gene whereas TaiN does not. S2 cells were transfected with indicated constructs and luciferase reporter gene, followed by dual luciferase assay. The data were quantified using an unpaired *t*-test. The results represented the mean± s.e.m. ***P<0.001, **P<0.01, n.s., no significant difference (*n*=3). Note that the activity of TaiC is even higher than that of Tai full length. (**c**) Schematic diagram of the different domains of Tai protein. (**d**) CoIP of Myc-tagged TaiP2, TaiP3, Tai-P5 and Flag-Yki. S2 cells were transfected with indicated constructs, followed by immunoprecipitation and western blot analysis with indicated antibodies. (**e**) Tai-P5 acts synergistically with Sd-Yki to activate *3xSd2-luc* reporter gene. Tai acts synergistically with Sd-Yki to activate *3xSd2-luc* reporter gene. S2 cells were transfected with indicated constructs and luciferase reporter gene, followed by dual luciferase assay. The data were quantified using an unpaired *t*-test. The results represented the mean± s.e.m. ***P<0.001, **P<0.01, *P<0.1, n.s., no significant difference (*n*=3). (**f**) Schematic diagram of the different variants of Tai protein C-terminus. (**g**) TaiC-ΔP3 enhances the transcriptional activity of Sd-Yki while TaiC-ΔP5 does not. The data were quantified using an unpaired *t*-test. The results represented the mean±s.e.m. ***P<0.001 (*n*=3), ***P<0.001, **P<0.01, *P<0.1, n.s., no significant difference (*n*=3).

We next mapped which domains of Tai mediate its function. Different truncations of Tai were generated and tested for their activities on promoting Sd-Yki transcriptional activity. Two Tai truncations were generated, namely TaiN (1-650aa) and TaiC (650aa-2036aa) (Figure 7a). In a dual luciferase assay, TaiN did not affect the transcriptional activity of Sd-Yki whereas TaiC significantly promoted the activity of Sd-Yki (Figure 7b). We also generated *UAS-Myc-TaiN* and *UAS-Myc-TaiC* transgenic flies. Consistent with the findings from experiments using S2 cells, coexpression with TaiN did not affect the eye overgrowth caused by *GMR-Yki* whereas coexpression of TaiC enhanced the *GMR-Yki*-induced phenotype (Supplementary Figure S2R-U). Of note, both *in vitro* and *in vivo* assays implicated that the function of TaiC is even stronger than that of Tai full length (Figure 7b and Supplementary Figure S2R-U). All these observations suggest that C-terminus of Tai is necessary and sufficient for the activity of Tai.

To further clarify the essential domains of Tai, the full-length protein was cut into smaller truncated forms (P1–P5) (Figure 7c). As shown in Figure 7d, TaiP2, TaiP3 and Tai-P5 were co-immunoprecipitated with Yki. However, only Tai-P5 significantly enhanced the activity of Sd-Yki (Figure 7e), suggesting that Tai-P5 is crucial for the function of Tai. To further distinguish the roles of TaiP3 and Tai-P5, we generated TaiC-ΔP3 and TaiC-ΔP5 (Figure 7f) to dissect their function on promoting the activity of Sd-Yki. As shown in Figure 7g, TaiC-ΔP3 still maintained the ability to enhance Sd-Yki transcriptional activity, while TaiC-ΔP5 lost most of its function on promoting Sd-Yki activity, indicating that Tai-P5 is indispensable for the function of Tai in Hippo signaling. We further made Tai-ΔP5 to check its function. Tai-ΔP5 can bind to EcR but does not facilitate Yki-Sd transcription activity, suggesting that the EcR-binding ability is dispensable for Tai’s function in activating Yki-Sd (Supplementary Figure S2V and W). In conclusion, the results presented above suggested that C-terminus of Tai is essential for the function of Tai in the Hippo pathway and Tai-P5 has an indispensable role in this process.

### Tai functions as a coactivator in both the Hippo pathway and the EcR pathway

Considering Tai is a multifunctional protein that was reported as a coactivator of EcR, it is an interesting question whether the function of Tai in the Hippo pathway is related to the EcR pathway. By genetic experiments, we found that, unlike depletion of Tai by RNAi, EcR RNAi neither changed the eye phenotype caused by Yki overexpression nor affected the expression of Hippo pathway target genes (Supplementary Figure S3A-D′). Meanwhile, we tested the function of the repressor of EcR signaling pathway, Abrupt (Ab), which antagonizes Tai to suppress EcR signaling. As shown in Supplementary Figure S3E-G′, the depletion of Ab by RNAi neither changed the eye phenotype caused by Yki overexpression nor affected the expression of Hippo pathway target genes. As EcR may be a default repressor in the absence of Tai, we also checked whether Tai depletion-induced phenotypes were caused by the loss of EcR. We took use of different EcR RNAi lines and found that expressing EcR RNAi transgenes could not rescue Tai RNAi-induced suppression of tissue growth (Supplementary Figure S3H-K). To further investigate the relationship between the Hippo pathway and the EcR pathway, we carried out *in vivo* staining assay and *in vitro* luciferase assay. The expression level of *Broad Complex Z3* (*BrC*), a classic EcR target gene, was significantly increased upon Tai overexpression, while the increase of its protein level was not affected by Yki RNAi (Supplementary Figure S3L-N′). In a following luciferase assay using a EcR pathway reporter (pIND/lacZ(+5XEGRE), EcR-luc), coexpression with Yki slightly increased the activity of Tai in promoting EcR pathway activity (Supplementary Figure S3O). In another luciferase assay using Sd reporter, coexpression of EcR and Ultra-spiracle (Usp) slightly inhibited the function of Tai in promoting Sd-Yki activity, suggesting that Sd-Yki and EcR-Usp may contend with each other for the co-factor Tai (Supplementary Figure S3P). Thus, we conclude that Tai may act as a coactivator for both Hippo and EcR signaling.

### Tai is required for ISC proliferation

**Figure 8:**
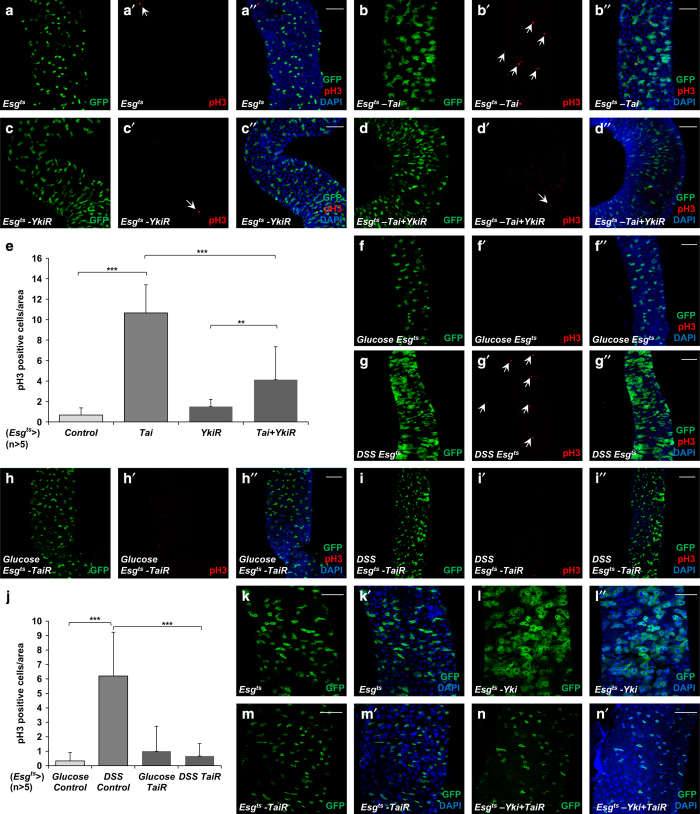
Tai is required for ISC proliferation. (**a**–**d**′′) Midguts from adult flies of indicated genotypes were stained with pH3 antibody (red) and DAPI. esg-GFP marked the ISCs/enteroblasts (EBs) and pH3-positive cells were indicated with arrows. Scale bars, 50 μm. (**e**) The comparison of the number of pH3-positive cells shown in **a**–**d**′′. The data were quantified using an unpaired *t*-test. The results represented the mean+s.e.m. ***P<0.001, **P<0.01, *P<0.1, (*n*>5) for each genotype. (**f**–**i**′′) Midguts from adult flies of indicated genotypes treated with glucose or DSS were stained with pH3 antibody (red) and DAPI. esg-GFP marked the ISCs/EBs and pH3-positive cells were indicated with arrows. Scale bars, 50 μm. (**j**) The comparison of the number of pH3-positive cells shown in **f**–**i**′′. The data were quantified using an unpaired *t*-test. The results represented the mean+s.e.m. ***P<0.001, **P<0.01, *P<0.1, (*n*>5) for each genotype. Scale bars, 50 μm. (**k**–**n**′) Midguts from adult flies of indicated genotypes were stained with DAPI. *esg*-GFP marked the ISCs/EBs. Scale bars, 50 μm.

Besides the roles of the Hippo pathway in growth control, it also has an essential role in regulating ISC proliferation. Thus, we investigated the function of Tai in ISC proliferation control. As shown in Figure 8a-b′′, the overexpression of Tai in ISCs/enteroblasts induced a significant increase of pH3-positive cell number, suggesting that Tai promotes ISC proliferation. Moreover, depletion of Yki by RNAi suppressed the upregulation of pH3-positive cell number induced by Tai overexpression, implying that this function of Tai depends on Yki (Figure 8c-e).

DSS-induced tissue damage stimulates ISC proliferation in a Hippo pathway-dependent manner. To determine whether Tai participates in the regulation of tissue damage-induced ISC proliferation, flies expressing Tai RNAi in ISCs/enteroblasts were treated with DSS. As shown in Figure 8f-j, Tai RNAi in ISCs/enteroblasts suppressed DSS-induced ISC proliferation, as indicated by the reduction in the number of esg-GFP positive and pH3-positive cells. These observations suggest that Tai is required for DSS-stimulated ISC proliferation. Furthermore, we tested whether Tai is required for Yki-induced ISC proliferation. Tai RNAi significantly reduced the esg-GFP-positive cell number induced by Yki overexpression, indicating that Tai RNAi inhibited Yki-induced ISC proliferation (Figure 8k-n′). All the results above suggest that Tai could sufficiently promote ISC proliferation and is required for both DSS-induced and Yki-induced ISC proliferation, which strengthen the assumption that Tai has an important role in the Hippo pathway. Moreover, EcR RNAi did not rescue the ISC over-proliferation induced by Tai, suggesting that Tai regulates ISC proliferation in a Yki-dependent manner rather than an EcR-dependent manner (Supplementary Figure S4A-E).

## Discussion

The Hippo signaling pathway is a central and highly conserved pathway that controls tissue growth and organ size. Although many detailed regulation mechanisms of several core Hippo pathway components have been defined, the mechanism by which Sd-Yki activates target gene transcription is not completely understood. A study published recently by Zhang *et al*. [19] addressed that Tai and Yki collaboratively induce germline genes in developing somatic epithelia to regulate tissue growth. Here, we independently provide both genetic and biochemical evidence that Tai functions as a coactivator of Yki and facilitates the activity of Yki-Sd to activate the transcription of several Hippo pathway target genes. Moreover, we found that the C-terminus of Tai is essential for its function in the Hippo pathway. Furthermore, we demonstrated that Tai has an essential role in *Drosophila* ISC proliferation in a Yki-dependent manner.

Zhang *et al*. showed that Yki and Tai/EcR regulate canonical Hippo targets (diap1, ex and bantam) in parallel at endogenous Yki activity level [19]. In our hands, Tai was indispensable for Yki-induced upregulation of DIAP1 (Figure 5c-f′) and *bantam* microRNA (Supplementary Figure S2F-H′). Luciferase assay results indicated that Tai activated Yki-Sd complex, and EcR/Usp seems to contend for Tai with Yki-Sd complex (Supplementary Figure S3P). So, our findings demonstrated that Tai is indispensable for Yki's function in regulating some canonical Hippo targets transcription (such as *diap1* and *bantam*) even at endogenous Yki activity level.

Another interesting finding of our study is that the C-terminus of Tai has an essential role in the function of Tai and Tai-P5 is indispensable. It should be mentioned that, although Tai-P5 enhanced the transcriptional activity of Sd-Yki in S2 cells, neither Tai-P5 nor TaiC-ΔP5 could promote tissue growth as TaiC did (data not shown). Maybe, the integrity of the C-terminus of Tai is prerequisite for its activity. The newly published work by Zhang *et al.* demonstrated that Tai binds to Yki through two PPxY motifs that are indispensable for the function of Tai [19]. In our mapping experiment, TaiP2 and TaiP3 are still able to bind to Yki (Figure 7d). Maybe these binding sites were masked in the full-length Tai.

It is also worth noting that overexpression of Tai was sufficient to induce the upregulation of Hippo pathway target genes and enhance the tissue overgrowth caused by Yki, which distinguishes Tai as a potential oncogene. The mammalian homolog of Tai, AIB1, is reported as an oncogene that tightly correlated with a wide variety of human cancers [21–23]. It will be interesting to examine the functional relationship between AIB1 and the Hippo pathway in mammalian cells. Moreover, in this study, we also noticed that TaiC had a stronger function than full-length Tai. Coincidentally, an N-terminal truncated AIB1, AIB1-Δ3, was reported as a more active form, which is overexpressed in breast cancer cells and in breast tumor tissue [24]. Therefore, subsequent research on Tai and the Hippo pathway may help us to clarify the underlying mechanisms and provide excellent targets for the development of new cancer therapeutics.

## Materials and Methods

### Plasmids and cloning

*UAS-tai* plasmid was a gift from Denise Montell lab. Truncations of Tai (TaiN, TaiC, Tai-P1, TaiP2, TaiP3, Tai-P4, Tai-P5, TaiC-ΔP3, TaiC-ΔP5) were constructed according to a standard protocol. For expression in S2 cells and flies, constructs were cloned into the *pUAST-Myc* vector. All primers used in this study can be requested. Other constructs used in this work are as follows: *pUAST-Flag-Yki, pUAST-HA-SD, pIND/lacZ(+5XEGRE), pUAST-Flag-Hpo, pUAST-EcR.B and pUSAT-Usp.*

### Drosophila genetics

To make *tai* mutant fly stock, the CRISPR-Cas9 system was used to generate *taiD3* allele following the previously described protocol. Tai RNAi fly stocks were generated according to the procedure described in the study by Wang *et al.* [25]. Fly stocks used in this study are as follows: *UAS-Tai*, *UAS-Myc-TaiN*, *UAS-Myc-TaiC*, *tai*^*61G1*^ (from Montell lab, University of California, Santa Barbara, CA, USA), *taiD3*, *actin>CD2>Gal4, UAS-Tai-RNAi, Tai-RNAi* (VDRC NO.15709, Vienna, Austria)*, UAS-Yki, UAS-Myc-YkiS168A, UAS-YkiRNAi, UAS-EcR.A-IR* (Bloomington NO.9328, Bloomington, IN, USA)*, EcRRNAi* (Bloomington NO.29374, NO. 50712, NO.58286), *UAS-AbruptRNAi* (Bloomington NO.29407, NO.32378), *UAS-SdRNAi, wts*^*latsX1*^ *, sd*^*1*^ *, hh-Gal4, GMR-Gal4, MS1096-Gal4, act>CD2>Gal4, eyless-Gal4, diap1-lacZ, diap3.5-GFP, ex-lacZ, bantam-GFP, eyflp; FRT40, hsflp;FRT40 M(2)24F arm-lacZ* (from Jiang Lab, The University of Texas Southwestern Medical Center, TX, USA)*, hsflp actin-Gal4 UAS-GFP;FRT40 tubGal80*, *hsflp actin-Gal4 UAS-GFP; FRT82 tubGal80, esgGal4; tubGal80*^*ts*^.

All genetic experiments were conducted at 25 °C unless otherwise indicated. FLP/FRT-mediated mitotic recombination was induced 3 days after eggs were laid. To this end, the larvae were incubated at 37 °C for 30 min to generate mutant clones. To induce conditional expression in adult flies, *tub-Gal80ts* flies were maintained at 18 °C though development, and then 2–6-day-old adult females were transferred to 29 °C for 7–9 days before dissection.

### Cell culture, transfection, immunoprecipitation, western blot analysis, luciferase reporter assay and immunostaining

S2 cells were cultured in *Drosophila* Schneider’s Medium (Invitrogen, Carlsbad, CA, USA) with 10% heat-inactivated fetal bovine serum, 100 U ml^−1^ of penicillin, and 100 mg ml^−1^ of Streptomycin at 25 °C. Cell transfection was performed using LipofectAMINE (Invitrogen, cat. no. 11668-019) according to the manufacturer’s instructions. The cells were harvested 48 h after transfection. For the luciferase reporter assay, the *3xSd2-Luc* reporter system has been carried out as described [26]. For the EcR luciferase reporter assay, S2 cells were transfected with pIND/lacZ(+5XEGRE) (the reporter), Renilla (the internal control) and other indicated plasmids. Twelve hours after transfection, cells were treated with dimethyl sulfoxide or 3 μM Muriateron A as indicated. Cells were harvested 24 h after treatment and assayed using the Dual Luciferase reporter assay system according to the manufacturer’s instructions (Promega, Madison, WI, USA, cat. no. E1960).

The luciferase assay was performed using the Dual Luciferase Assay System (Promega, cat. no. E1960). Immunostaining and confocal microscopy were performed according to standard protocols. BrdU assays were performed as described previously [9].

The following antibodies were used in this work: Rat anti-Tai (1:500; from Montell Lab), Rabbit anti-Tai (1:500; generated by Shanghai Immune Biotech CO., Ltd. (Shanghai, China) against TaiN and TaiC separately), mouse anti-Flag antibody (1:5 000; Sigma, St Louis, MO, USA, cat. no. M8823), mouse anti-Myc antibody (1:5 000; Santa Cruz, Santa Cruz, CA, USA, cat. no. sc-40), mouse anti-HA antibody (1:5 000; Sigma, cat. no. H3663), Rat anti-Cubitus interruptus (Ci) antibody (1:250; Developmental Studies Hybridoma Bank, DSHB, University of Iowa, Iowa City, IA, USA, cat. no. 2A1), rabbit anti-PH3 (1:200; Cell Signaling, Danvers, MA, USA, cat. no. 9701), mouse anti-BrC-Z3 (1:200; DSHB), Rabbit anti-lacZ antibody (1:500; Invitrogen, cat. no. A-11132), Rabbit anti-Ex (1:50), mouse anti-DIAP1 (1:50; a gift from Bruce A Hay, California Institute of Technology), mouse anti-BrdU (1:100; Abcam, Cambridge, UK, cat. no. ab6326).

Images of cells, imaginal discs and guts were acquired with a confocal microscope (LAS SP5; Leica, Wetzlar, Germany) using a ×20/1.25 NA oil objective (Leica) or ×40/1.4 NA oil objective (Leica) at room temperature. To reduce the variables in experiments involving comparison between different genotypes, all samples were stained, processed and imagined in parallel. Images were then processed, scaled and analyzed using Adobe Photoshop (Adobe, San Jose, CA, USA).

### ChIP-PCR, DNA pull-down and real-time PCR

For ChIP, S2 cells were transfected with *UAS-HA-Yki*, *UAS-Myc-Tai* and *UAS-HA-Sd*. Forty-eight hours later, cells were cross-linked in 1% formaldehyde, and quenched in 125 mM glycine. Lysates were prepared in lysis buffer (50 mM HEPES-KOH pH 7.5, 140 mM NaCl, 1 mM EDTA pH 8.0, 1% Triton X-100, 0.1% sodium deoxycholate, 0.1% SDS and protease inhibitors (Sigma, cat. no. P8340), followed by sonication prior to centrifugation. Indicated antibodies and Protein A/G agarose beads (Santa Cruz, cat. no. sc2003) were mixed with lysates and then washed (0.1% SDS, 1% Triton X-100, 2 mM EDTA pH 8, 150 mM NaCl, 20 mM Tris-HCl pH 8), eluted (50 mM NaHCO_3_, 1% SDS), extracted and suspended (in TE buffer). PCR primers are as follows: th1-F (5′-CAGTATACATACTTCTGC-3′), th1-R(5′-TTGATGGTAGGATGACAC-3′), th2-F (5′-GCTATTATATTATTGTG-3′), th2-R (5′-GGATTATGAGTGTGTGCG-3′), thB-F (5′-GATTTTATAATCTTATCG-3′), thB-R (5′-GCCTCCAGATTGTTTTAG-3′).

For DNA pull-down, S2 cells were transfected with *UAS-HA-Yki*, *UAS-Myc-Tai* and *UAS-HA-Sd* and then collected. The following procedure was performed as previously described [13].

For real-time PCR, RNA was isolated from wing discs. Coding DNA was synthesized with cDNA synthesis kit (Toyobo, cat. no. FSQ-301). RT-PCR was performed on ABI Fast 7500 using SYBR Green (Toyobo, Shanghai, China, cat. no. QPK-201). Rp49 was used as normalization control. Primers for real-time PCR are list as following: rp49-F(5′-CGGATCGATATGCTAAGCTGT-3′); rp49-R(5′-CGACGCACTCTGTTGTCG-3′); EcR-F (5′-AAGGAGAAGGACAAAATG-3′); EcR-R (5′-GTCATAAGGTCAAGAATCT-3′).

### Statistical analysis

All statistical data were analyzed using Student's *t*-test by R 2.9.0 and expressed as mean±s.e.m. Results were considered statistically significant when *P*<0.05.
