# Supplementary material for: Taiman acts as a coactivator of Yorkie in the Hippo pathway to promote tissue growth and intestinal regeneration
Source: Cell Discov. 2016 Mar 22;2:16006–. doi: 10.1038/celldisc.2016.6 (PMC4860958; doi:10.1038/celldisc.2016.6)
Supplement: Supplementary Figure S1 [file celldisc20166-s1.pdf]

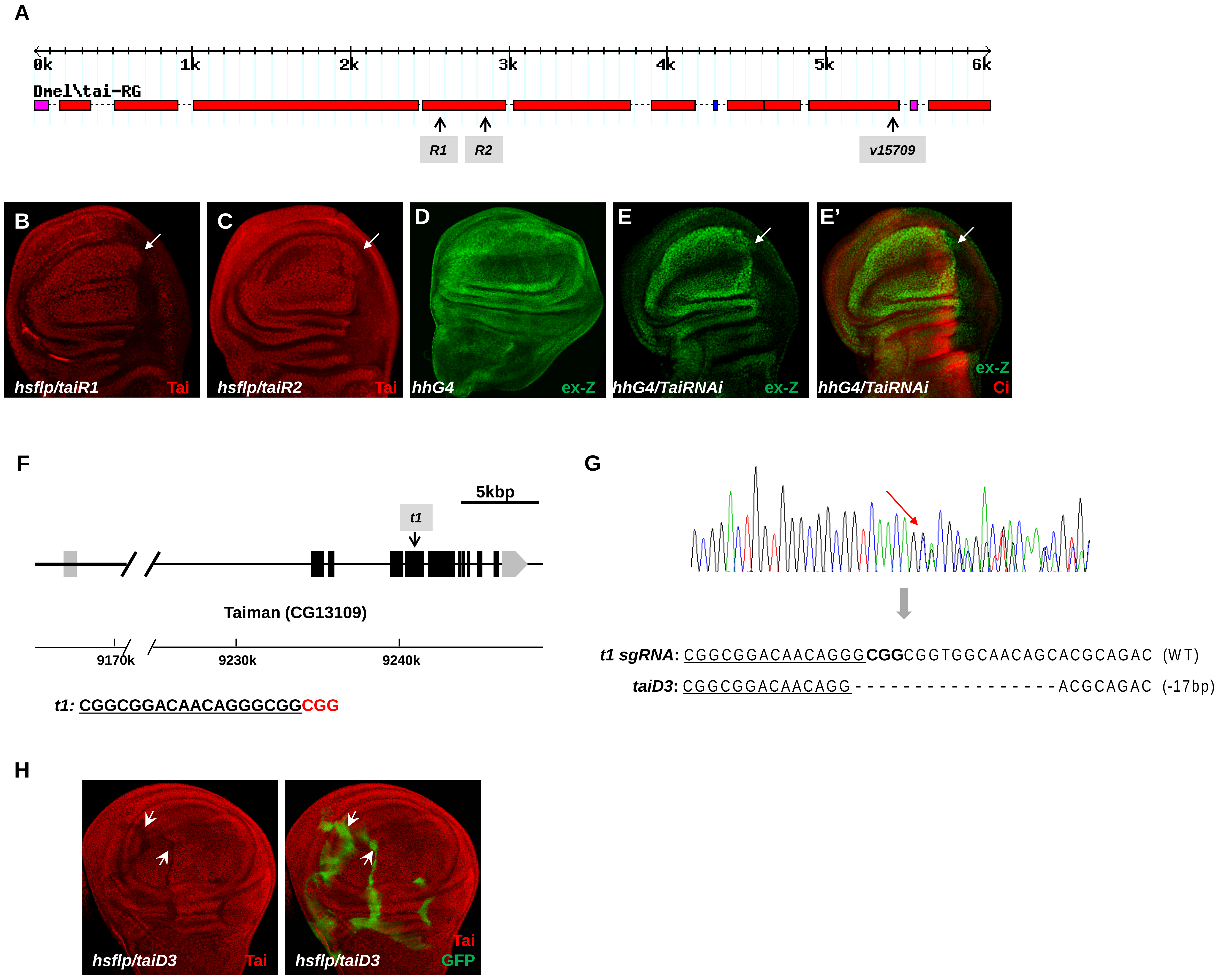

Supplementary Figure 1. Tai RNAi and *taiD3* mutation.
(A) Diagram of the target regions of different *Tai-RNAi* used in this study. The schematic diagram is referred to Flybase website. (B, C) Wing discs expressing newly made *Tai-RNAi* transgenes under the control of *hhGal4* were stained with indicated antibodies. Arrows indicate the P-compartment. Scale bars, 100µm. (D-E') Control wing discs (D) or wing discs expressing *Tai-RNAi* with *hh-Gal4* (E-E') were immunostained to show the expression of *ex-lacZ*. Arrows indicate the P-compartment. Scale bars, 100µm. (F) Schematic diagram of the target site (*t1*) of CRISPR/Cas9 system and the target DNA sequence. *t1* target site localized in the fourth exon of *tai* gene and was indicated by the arrow. The target DNA sequence was indicated with underline, the protospacer adjacent motif (PAM) in red. (G) Mutation in *taiD3* variant. The CRISPR/Cas9 system induced a 17bp deletion in the target region. The sequencing result showed double peaks which was marked by the red arrow, indicating the generation of mutation in the *tai* gene. Deleted bases are marked with dashes. (H) Wing discs containing *taiD3* mutant clones were stained with Tai antibody. Clones are marked by GFP and indicated by arrows. Scale bars, 100µm.
