# Supplementary material for: Taiman acts as a coactivator of Yorkie in the Hippo pathway to promote tissue growth and intestinal regeneration
Source: Cell Discov. 2016 Mar 22;2:16006–. doi: 10.1038/celldisc.2016.6 (PMC4860958; doi:10.1038/celldisc.2016.6)
Supplement: Supplementary Figure S2 [file celldisc20166-s2.pdf]

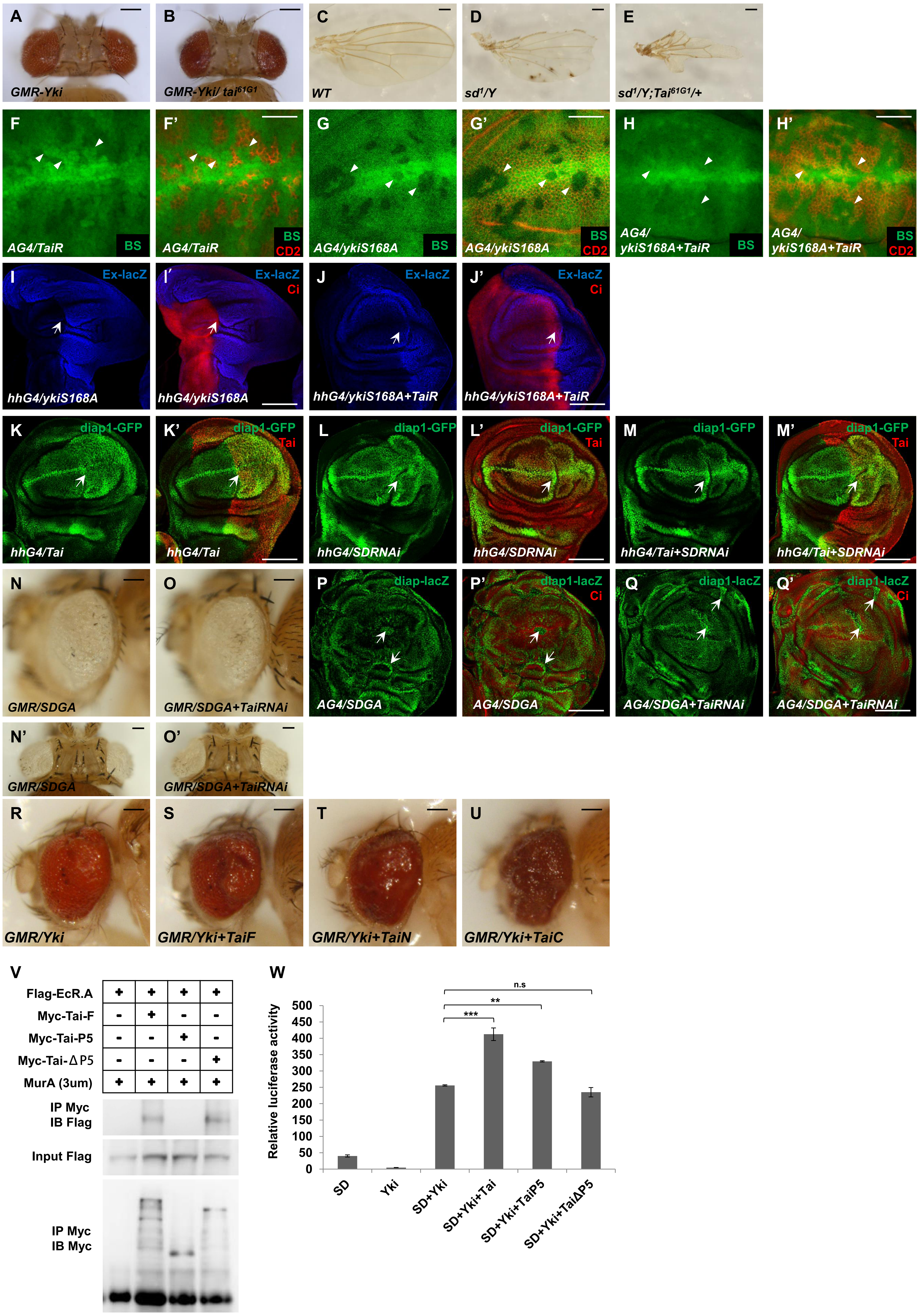

Supplementary Figure 2. Genetic interaction analysis and TaiC function.

(A, B) Side views of the adult flies with indicated genotypes. *GMR-Yki* (A), *GMR-Yki/tai^61G1^* (B). Scale bars, 100µm. (C-E) Side views of the adult flies with indicated genotypes. Scale bars, 250µm. (F-H') *Drosophila* discs containing flip-out clones expressing *TaiRNAi* (F,F') *or ykiS168A* (G,G') *or ykiS168A+TaiRNAi* (H,H') under the control of *act>CD2>Gal4* (*AG4*) were immunostaind to show the expression of *bantam-GFP* (*BS*). Clones were marked by the lack of CD2 staining and indicated by arrowheads. Scale bars, 100µm. (I-J') Tai RNAi could not suppressed the increase of ex-lacZ level induced by Yki. Wing discs expressing *ykiS168A* (I, I') or *ykiS168A+TaiRNAi* (J, J') under the control of *hh-Gal4* were immunostained to show the expression of *ex-lacZ*. P-compartment is marked by the overexpressed Tai and indicated by the arrows. Scale bars, 100µm. (K-M') Wing discs expressing *Tai* (K, K') or *SdRNAi* (L, L') or *Tai+SdRNAi* (M, M') under the control of *hh-Gal4* were immunostained to show the expression of *diap1-GFP*. P-compartment is marked by the overexpressed Tai and indicated by the arrows. Scale bars, 100µm. (N-O') Side (N, O) and dorsal (N', O') views of eyes expressing *SdGA* (N, N') or *SdGA+TaiRNAi* (O, O') with *GMR-Gal4*. Scale bars, 100µm. (P-Q') *Drosophila* discs containing flip-out clones expressing *SdGA* (P, P') or *SdGA+TaiRNAi* (Q, Q') by *act>CD2>Gal4* were immunostaind to show the expression of *diap-lacZ*. Clones were indicated by the lack of CD2 staining and arrowheads. Scale bars, 100µm. (R-U) Side views of eyes expressing *Yki* (R), *Yki+Tai* (S), *Yki+TaiN* (T) or *Yki+TaiC* (U) with *GMR-Gal4*. Scale bars, 100µm. (V) CoIP of Myc-tagged Tai, Tai-P5, Tai-ΔP5 and Flag-EcR.A. S2 cells were transfected with indicated constructs, followed by immunoprecipitation and western blot analysis with indicated antibodies. (W) Tai or Tai-P5 acts synergistically with Sd-Yki to activate *3xSd2-luc* reporter gene while Tai-ΔP5 could not. S2 cells were transfected with indicated constructs and luciferase reporter gene, followed by dual luciferase assay. The data was quantified using an unpaired t-test. The results represented the mean± SEM. *** means p<0.001, ** means p<0.01, * means p<0.1, n.s means no significant difference (n=3).
