# Supplementary material for: Taiman acts as a coactivator of Yorkie in the Hippo pathway to promote tissue growth and intestinal regeneration
Source: Cell Discov. 2016 Mar 22;2:16006–. doi: 10.1038/celldisc.2016.6 (PMC4860958; doi:10.1038/celldisc.2016.6)
Supplement: Supplementary Figure S3 [file celldisc20166-s3.pdf]

Supplementary Figure 3 *Wang et al.*

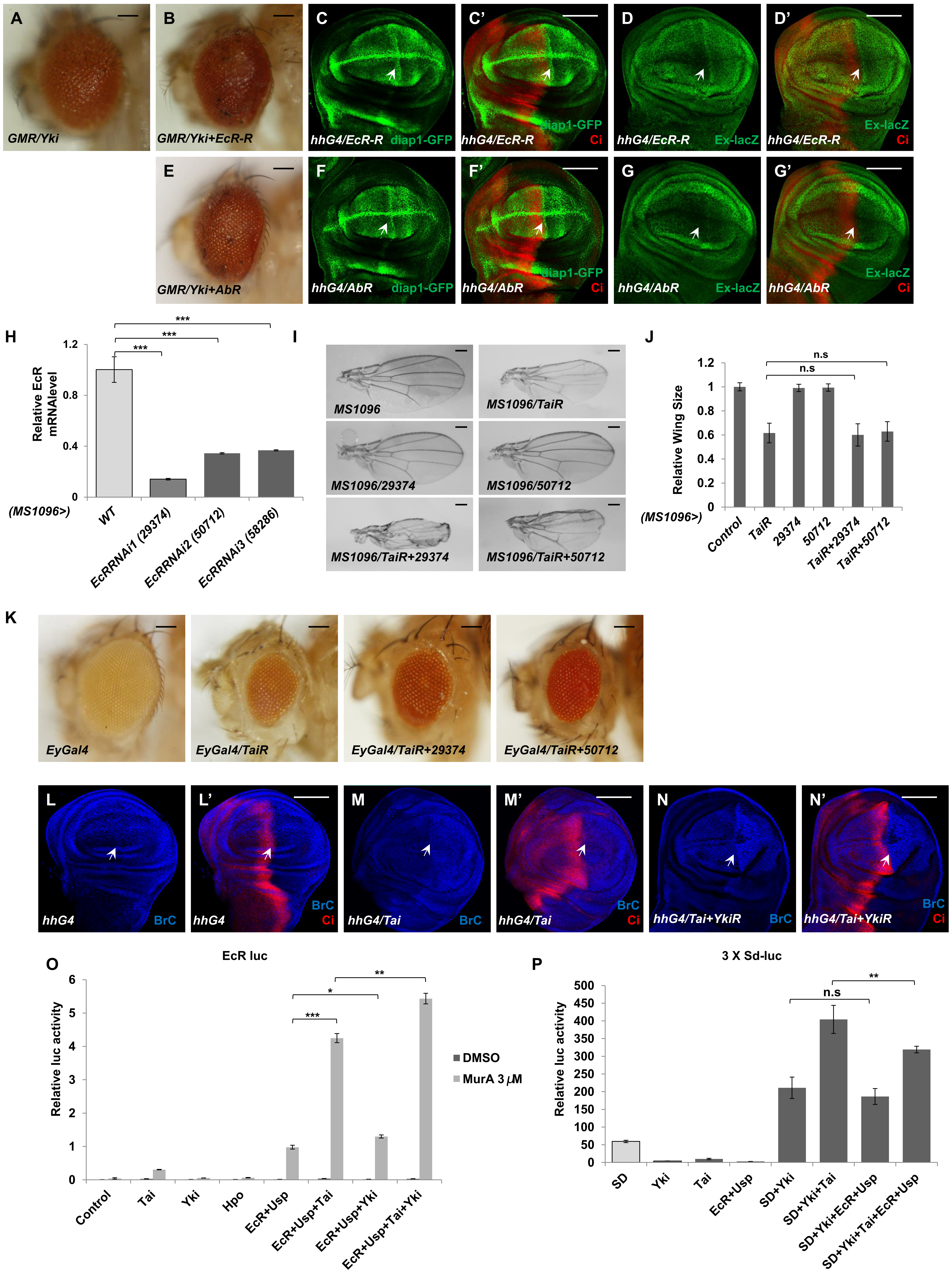

Supplementary Figure 3. Tai functions in both Hippo pathway and EcR pathway.
(A, B) Side views of the adult flies with indicated genotypes. *GMR-Yki* (A), *GMRYki+EcRRNAi (EcR-R)* (B). Scale bars, 100µm. (C-D') Wing discs expressing EcR-R under the control of *hh-Gal4* were immunostained to show the expression of *diap1-GFP* (C, C') or *ex-lacZ* (D, D'). P-compartment is marked by the overexpressed Tai and indicated by the arrows. Scale bars, 100µm. (E) Side views of the adult flies of *GMR-Yki+AbruptRNAi (Ab-R)*. Scale bar, 100µm. (F-G') Wing discs expressing Ab-R under the control of *hh-Gal4* were immunostained to show the expression of *diap1-GFP* (F, F') or *ex-lacZ* (G, G'). P-compartment is marked by the overexpressed Tai and indicated by the arrows. Scale bars, 100µm. (H) Real-time PCR to check RNAi efficiency of different *EcR RNAi* lines. Different *EcR RNAi* lines were crossed with *MS1096-Gal4* and larval wing discs were dissected to verify EcR mRNA level through Real-time PCR. (I) Dorsal views of adult wings of the indicated genotypes. Scale bars, 100µm. (J) The relative wing size of different genotypes showed in (I). The data was quantified using an unpaired t-test. The results represented the mean± SEM. *** means $p<0.001$ ($n>6$) for each genotype. (K) Side views of adult eyes of the indicated genotypes. Scale bars, 100µm. (L-N') Wing discs of *hh-Gal4* (L, L') or expressing *Tai* (M, M') or *Tai+YkiRNAi* (N, N') under the control of *hh-Gal4* were immunostained to show the expression of BrC. P-compartment is marked by the overexpressed Tai and indicated by the arrows. Scale bars, 100µm. (O) Yki slightly promoted Tai to activate *EcR-luc* reporter gene. S2 cells were transfected with indicated constructs and luciferase reporter gene and treated with DMSO or 3µM Muriateron A (MurA), followed by dual luciferase assay. The data was quantified using an unpaired t-test. The results represented the mean± SEM. *** means $p<0.001$, ** means $p<0.01$, * means $p<0.1$, n.s means no significant difference ($n=3$). (P) EcR/Usp slightly suppressed the activation of Sd/Yki induced by Tai. S2 cells were transfected with indicated constructs and luciferase reporter gene, followed by dual luciferase assay. The data was quantified using an unpaired t-test. The results represented the mean± SEM. *** means $p<0.001$, ** means $p<0.01$, * means $p<0.1$, n.s means no significant difference ($n=3$).
