# Supplementary material for: Taiman acts as a coactivator of Yorkie in the Hippo pathway to promote tissue growth and intestinal regeneration
Source: Cell Discov. 2016 Mar 22;2:16006–. doi: 10.1038/celldisc.2016.6 (PMC4860958; doi:10.1038/celldisc.2016.6)
Supplement: Supplementary Figure S4 [file celldisc20166-s4.pdf]

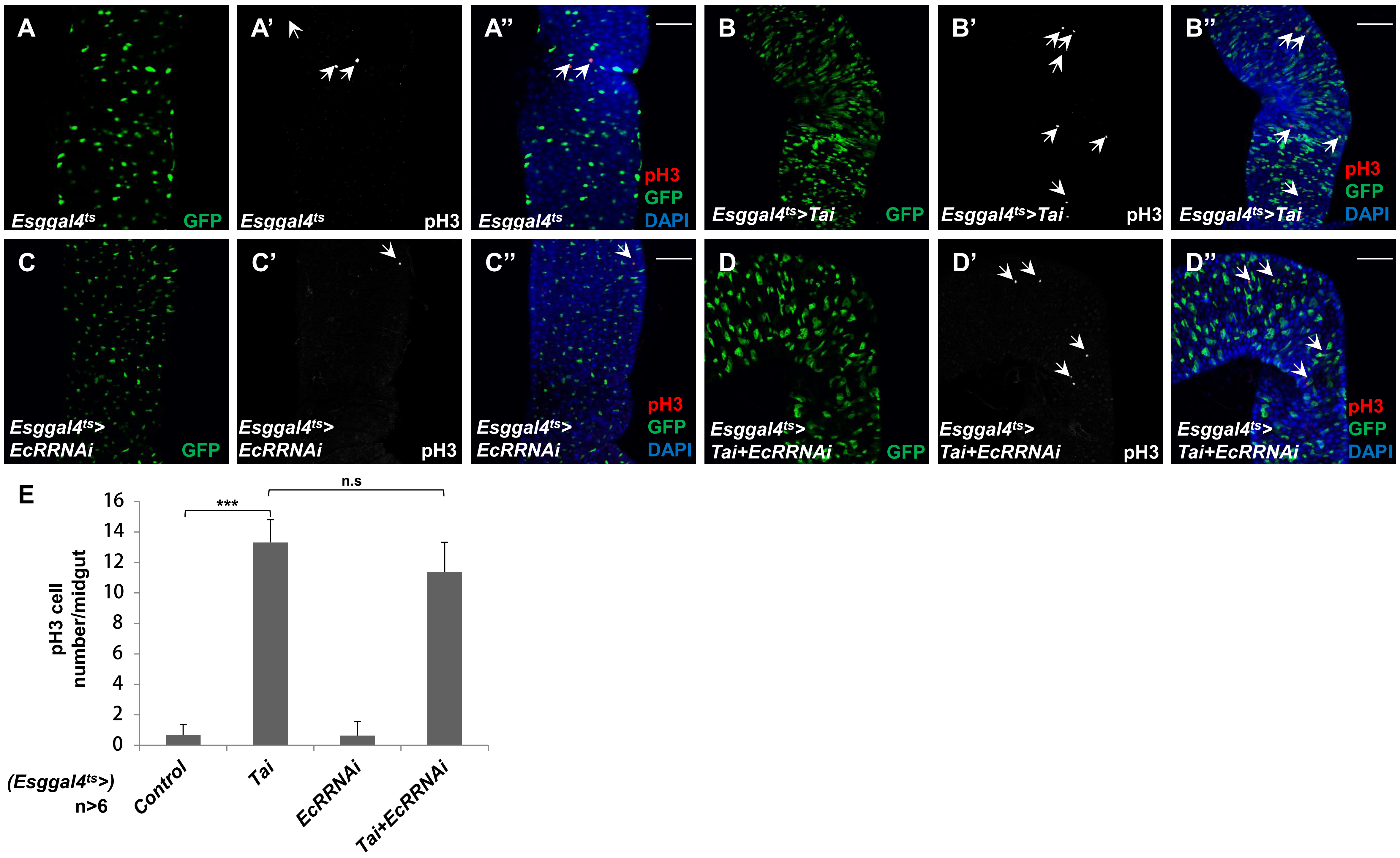

Supplementary Figure 4. EcRRNAi could not rescue the ISC over-proliferation induced by Tai in fly adult midgut.
(A-D") Midguts from adult flies of indicated genotypes treated with Glucose or DSS were stained with pH3 antibody (Red) and DAPI. esg-GFP marked the ISCs/EBs and pH3-positive cells were indicated with arrows. Scale bars, 50 µm. (E) The comparison of the number of pH3 positive cells shown in A-D". The data was quantified using an unpaired t-test. The results represented the mean+SEM. *** means p<0.001, ** means p<0.01, * means p<0.1, (n>6) for each genotype.
